# Duffy and Kidd Genotyping Facilitates Pretransfusion Testing in Patients Undergoing Long-Term Transfusion Therapy

**DOI:** 10.4274/tjh.2013.0075

**Published:** 2014-12-05

**Authors:** Diana Remeikiene, Rasa Ugenskiene, Arturas Inciura, Aiste Savukaityte, Danguole Raulinaityte, Erika Skrodeniene, Renata Simoliuniene, Elona Juozaityte

**Affiliations:** 1 Lithuanian University of Health Sciences, Institute of Oncology, Department of Haematology, Kaunas, Lithuania; 2 Lithuanian University of Health Sciences, Institute of Oncology, Oncology Research Laboratory, Kaunas, Lithuania; 3 Lithuanian University of Health Sciences, Institute of Oncology, Department of Oncology, Kaunas, Lithuania; 4 Lithuanian University of Health Sciences, Department of Laboratory Medicine, Kaunas, Lithuania; 5 Lithuanian University of Health Sciences, Department of Physics, Mathematics, and Biophysics, Kaunas, Lithuania

**Keywords:** Duffy phenotyping, Kidd phenotyping, Genotyping, Multitransfused patients

## Abstract

**Objective:** Conventional serologic typing of red blood cell systems other than ABO and RhD can be inaccurate and difficult to interpret in patients who have recently undergone blood transfusion. While molecular-based assays are not used routinely, the usefulness of genotyping was investigated in order to determine patients who may benefit from this procedure.

**Materials and Methods:** Blood samples were taken from 101 patients with haemato-oncological, chronic renal, or gastroenterological diseases and from 50 donor controls; the samples were tested for Fya and Fyb by applying serologic and genetic methods. All patients had received 3 or more units of RBCs during the last 3 months. An average of 6.1 RBC units were transfused per patient. The average length of time from transfusion until blood sampling was 24.4 days. The haemagglutination test was applied for serological analysis, and the restriction length polymorphism assay was used for genotyping.

**Results:** In total, 33 (32.7%) patients showed positive reactions with anti-Fya or anti-Fyb while being negative genetically. False-positive Fya results were found in 23 samples, and false-positive Fyb in 10 specimens. During the last 3 months, significantly more RBC units were transfused to patients with discrepant results than to those with accurate phenotyping/genotyping results: median of 5 (mean ± SE: 6.85±0.69) versus median of 4 (mean: 5.71±0.51), respectively (p=0.025). The median length of time after the last transfusion was 25 days (mean: 28.72±2.23 days) in the group with accurate phenotyping/genotyping results versus a median of 14 days (mean: 15.52±1.95 days) in the group with discrepant results (p=0.001). Phenotypes and genotypes coincided in all donor samples.

**Conclusion:** Genotyping assays for the Duffy system should be considered if the patient underwent blood transfusion less than 3 or 4 weeks before the sample collection. If the time frame from RBC transfusion exceeds 6 weeks, Duffy phenotyping can provide accurate results.

## Abstract (tr)

**Amaç:** Eritrositlerin ABO ve RhD sistemleri dışındaki konvansiyonel serolojik tiplendirmesini son zamanlarda sık kan transfüzyonu yapılmış olan kişilerde yorumlamak yanlış ve zor olabilir. Bazı moleküler incelemeler henüz rutin olarak kullanılmamasına rağmen, genotiplendirmenin yararı bu işlemden fayda görecek hastaları belirlemek amacıyla incelenmiştir.

**Gereç ve Yöntemler:** Hemato-onkoloijk, kronik böbrek hastalığı veya gastrointestinal hastalığı olan 101 hastadan ve kontrol grubu olan 50 kişiden karşılaştırmak üzere kan örnekleri alındı. Numuneler serolojik ve genetik yöntemler kullanılarak Fya ve Fyb için test edildi. Bütün hastalara son 3 ay içinde 3 ve daha fazla ünite eritrosit süspansiyonu transfüzyonu yapılmıştı. Her hasta için ortalama transfüzyon 6,1 ünite olarak hesaplandı. Transfüzyondan örneklerin alınmasına kadar geçen ortalama süre 24,4 gündü. Hemaglütinasyon testi serolojik analiz için uygulandı, ve uzunluğu kısıtlanmış polimorfizm testi genotipleme için kullanıldı.

**Bulgular:** Genetik olarak negatif olan, toplam 33 (%32,7) hastada anti-Fya veya anti-Fyb ile pozitif reaksiyon elde edildi. Yirmi üç örnekte yanlış pozitif Fya sonucu, 10 örnekte yanlış pozitif Fyb sonucu elde edildi. Son 3 ayda fenotiplendirme/genotiplendirme sonuçları tutarsız olanlarda uyumlu olanlara göre anlamlı olarak daha fazla eritrosit süspansiyonu transfüzyon yapılmıştı: sırasıyla ortanca 5 (ortalama ± SE: 6,85±0,69) ve ortanca 4 (ortalama ± SE: 5,71±0,51) (p=0,025). Fenotiplendirme/genotiplendirmesi sonuçları uyumlu olan grupta transfüzyondan test aşamasına kadar geçen ortanca süre 25 gün (ortalama: 28,72±2,23) iken, sonuçları uyumsuz çıkan grupta bu süre ortanca 14 (ortalama: 15,52±1,95) gündü. Tüm donör örneklerinde fenotip ve genotipler tutarlıydı.

**Sonuç:** Örnek alınmasından 3 veya 4 hafta öncesinde transfüzyon alan kişilerde Duffy sistemi için genotiplendirme yapılması uygun olabilir. Eğer eritrosit süspansiyonu transfüzyonundan sonra geçen süre 6 haftadan fazla ise, Duffy fenotiplendirmeyle uygun ve güvenilir sonuçlar sunabilir.

## INTRODUCTION

Patients who require multiple transfusions of red blood cells (RBCs), such as those with sickle cell disease (SCD) or β-thalassaemia, have a higher potential risk of alloimmunisation and delayed haemolytic transfusion reactions (DHTRs). The widely described risk of developing antibodies, mostly to Rh, Kell, Duffy, Kidd, and MNS systems, ranges from 18% to 47% [1,2]. Programs to prevent alloimmunisation have been implemented in the centres treating patients with SCD and β-thalassaemia [1,3,4]. In addition to ABO and RhD matching, protocols range from providing limited antigen-matched RBCs for Rh and Kell to extended antigen-matched RBCs for Rh, Kell, Duffy, Kidd, and MNS systems prior to transfusion.

Accurate phenotyping of multitransfused patients is often complicated, mostly due to the presence of circulating transfused donor RBCs in the recipient’s blood, leading to discrepancies in the assessment of tests results. The importance of the genotyping of clinically relevant antigens (such as C, c, E, K, Fya, Fyb, Jka, Jkb, and S) in addition to phenotyping is still being discussed for patients with SCD, β-thalassaemia, and other haemoglobinopathies.

However, there is a lack of information about the need for molecular testing for other groups of patients who depend on long-term RBCs transfusions, such as those with myelodysplastic syndrome, myelofibrosis, or chronic renal failure [5,6,7]. Delayed haemolytic transfusion reactions are often an issue in these patients, affecting their quality of life and sometimes being fatal. The selection of antigen-negative RBCs in order to reduce alloimmunisation is often required for patients with long-term transfusions and for those with formed alloantibodies.

The most common causes of DHTR include antibodies against Rh, Kell, Kidd, Duffy, and MNS systems [3,8,9,10]. Serological testing and evaluation of the antigens and antibodies of the Duffy and Kidd systems are among the main problems in multitransfused patients. The correlation between serological and molecular typing of Duffy and Kidd systems demonstrates the benefits of genotyping in patients who depend on chronic RBC transfusions.

The aim of our study was to estimate the value of DNA-based typing of Duffy and Kidd systems in chronically transfused non-SCD or β-thalassaemia patients, and to establish the impact of the amount of transfused RBCs and the time from the last transfusion on the discrepancy of the results.

## MATERIALS AND METHODS

**Patients**

Peripheral blood samples were obtained from 101 patients with haematological and oncological diseases, chronic renal failure, and gastrointestinal diseases. All patients received 3 or more units of RBCs during the last 3 months. The inclusion criteria were the time frame from the last RBC transfusion being shorter than 8 weeks and the need for further transfusions. None of the patients were tested for Fya, Fyb, Jka, or Jkb antigens before transfusions.

Most of the RBC units received by the haematology patients and some of those received by the oncology patients were leucodepleted, while others were non-leucodepleted. Thus, 62 patients (61.4%) were transfused with leucoreduced RBCs, and 39 (38.6%) with entirely (or by more than half) non-leucoreduced RBCs. The median number of transfusion events per patient was 3 (mean: 3.33±0.21, range: 1-12). A median of 2 (mean: 1.85±0.04, range: 1-4) RBC units were administered per transfusion event and 4 (mean: 6.1±0.41, range: 3-24) units per patient.

**Donors**

In total, 49 blood donors served as controls in our study. Blood samples of donors were obtained by taking 1 or 2 segments of tubes from RBC bags used for transfusions. This control group and the patient group were used to determine frequencies of Duffy and Kidd phenotypes in a Lithuanian population.

Blood samples of all patients and donors were tested for Fya, Fyb, Jka, and Jkb antigens by applying serological and molecular methods. Approval for the study was obtained from the Regional Bioethics Committee.

**Serotyping**

Peripheral blood samples were used for Duffy and Kidd phenotyping. Fya, Fyb, Jka, and Jkb antigens were determined by haemagglutination using anti-Fya, anti-Fyb, anti-Jka, and anti-Jkb Coombs reactive (polyclonal, human) reagents (Antitoxin GmbH, Germany) and DG Gel Coombs cards (Diagnostic Grifols, S.A., Spain). Each microtube of the card contained polymerised dextran in a buffered medium containing preservatives and low ionic strength solution, and was mixed with polyspecific anti-human globulin. The tests were performed according to the manufacturer’s recommendations. The results of the agglutination were expressed by using plus/minus values. Positive results were evaluated from 1+ to 4+.

**Genotyping**

The DNA was extracted from peripheral blood leucocytes using a DNA extraction kit (GeneJet Genomic DNA Purification Kit, Thermo Fisher Scientific, USA), following the manufacturer’s instructions. Polymorphism was identified by polymerase chain reaction-restriction length polymorphism (PCR-RFLP) analysis according to Reid et al. [7]. Briefly, each PCR reaction was carried out in a total volume of 25 µL containing 1X DreamTaq standard buffer, template DNA, 50 pM of each primer, 2.0 mM MgCl2, 200 µM of each dNTP, and 1 U of DreamTaq DNA polymerase (Thermo Fisher Scientific) with annealing at 62 °C.

The amplification products were then digested overnight by restriction endonuclease BanI (Thermo Fisher Scientific), following the manufacturer’s instructions. The fragments were separated electrophoretically using 2% agarose gel containing ethidium bromide.

The Duffy antigen is present in 2 major allelic forms, FY*A and FY*B, differing in an amino acid at position 42 (Gly42Asp) of the Duffy antigen receptor. The amino acid change occurs because of G125A polymorphism in the Duffy antigen receptor for the chemokine gene (DARC). 

The Kidd antigen system is known to comprise 2 major alleles, JK*A and JK*B, which result from a single nucleotide polymorphism (838G→A) in gene SLC14A1. The corresponding JK*A and JK*B antigens differ by a single amino acid (D280N).

**Statistical Analysis**

The comparison of medians between the groups was performed by applying the nonparametric Mann-Whitney U test. We did not compare the means because the values of the studied variables were not normally distributed (Kolmogorov-Smirnov test, p<0.05). The 2-proportion z-test, chi-square test, and Fisher’s exact test (for small samples) were used for categorical data analysis. The level of agreement between dichotomous results of 2 tests was measured using Cohen’s kappa. Differences were considered significant at p<0.05. IBM SPSS Statistics 20 was used for the data analysis.

## RESULTS

**Donors**

Coincidence between phenotype and genotype was observed in all 49 donor samples.

**Patients**

Disagreements of phenotype and genotype between the Duffy and Kidd systems were found in one-third of the samples of 101 patients who had recently undergone transfusion. The results of phenotyping and genotyping are presented in Table 1.

To analyse the impact of time from the last transfusion on the discrepancies of the results, patients were distributed into 2 groups. The first group included those who received RBCs less than 4 weeks before and the second group more than 4 weeks before the sample collection. The influence of the number of transfused RBCs on the disagreement of the results was also analysed.

**Duffy System**

A total of 33 (32.7%) phenotype/genotype discrepancies were assessed. However, in samples that did not contain one of the Duffy antigens, discrepant results were found in 68.8% of cases.

Significant differences in phenotype/genotype disagreements between the 2 aforementioned groups were found in 29 and 4 cases, respectively (Table 2).

The comparison of the duration of time after the last transfusion between the 2 groups of patients with accurate and discrepant phenotype/genotype results also showed a significant difference. The median of days after the last transfusion was found to be 14 (mean: 15.52±1.95) in the group with discrepant results versus 25 (mean: 28.72±2.23) in the group with accurate results (p=0.001).

No effect of transfused RBCs per year on Duffy phenotyping and genotyping discrepancies was detected: 605 units [median:5 (mean: 8.91±1.58, range: 3-46)] were transfused to patients with accurate phenotype/genotype results, and 294 units [median: 6 (mean: 8.91±1.4, range: 3-81] to those with discrepant results (p=0.207).

Significantly different results were found when comparing the number of RBC units transfused during the last 3 months between the 2 aforementioned groups. A median of 5 (mean: 6.85±0.69) units were transfused during this period to the patients with discrepant phenotype/genotype results versus 4 (mean: 5.71±0.5) to those with accurate results (p=0.025).

**Kidd System**

Disagreements between genotyping and serologic typing for Jka/Jkb were found in 32 (31.7%) blood samples, while in samples that did not contain one of the Kidd antigens, discrepant results were found in 65.3% of cases. 

Significantly more discrepant phenotype/genotype results were found in the samples of patients of the first group than in those of the second group, at 27 and 5 cases, respectively (Table 3).

The time after the last transfusion was shown to be significantly different between the 2 groups of patients. The median of days after the last transfusion was found to be 10.5 (mean: 16.09±2.45) in the group with discrepant results versus 25 (mean: 28.26±2.13) in the group with accurate results (p=0.01).

No effect of the transfused RBCs per year on Kidd phenotyping and genotyping discrepancies was detected: 649 units [median: 5 (mean: 9.41±1.66, range: 3-81)] were transfused to patients with accurate phenotype/genotype results, and 250 units [median: 8 (mean: 7.84±0.72, range: 3-18)] to those with discrepant results (p=0.188). There was no difference with regard to the number of RBC units transfused during the last 3 months between the 2 aforementioned groups, either. A median of 6 (mean: 6.28±0.48) units were transfused during this period to the patients with discrepant phenotype/genotype results versus a median of 4 (mean: 5.99±0.56) to those with accurate results (p=0.71).

**Phenotype Frequencies**

Genotyping results were used to determine the expected Duffy and Kidd phenotype frequencies. No differences were found when comparing these results with those of other authors [8,9]. The phenotypes of 101 patients and 49 controls are presented in Table 4.

## DISCUSSION

It is still disputable which situations require extended typing of transfused RBCs beyond routine matching for ABO, RhD, and existing antibodies. Although a number of studies found that SCD or β-thalassaemia patients may benefit from extended antigen matching, there is still a lack of recommendations in this context for other patients undergoing long-term RBC transfusions [1,11,12].

Additional antibodies are known to occur in 20% to 90% of previously alloimmunised patients [13,14,15]. Schonewille et al. evaluated the incidence of new antibody formation with subsequent transfusion in 22% of alloimmunised haemato-oncology patients and in 20%-25% of the non-haematology/oncology cohort [14,15]. The transfusion of extended antigen-matched RBCs, including Duffy and Kidd systems, has been shown to lower alloimmunisation in about 70% of cases [16,17]. According to these studies as well as those investigating SCD patients, extended antigen matching could be recommended for haemato-oncology patients and other subjects who undergo long-term transfusions.

We agree with the many authors who state that antigen typing of recently transfused patients is not always accurate, as their peripheral blood contains transfused cells [5,6,13,18,19]. Several reports describe this type of discrepancy in up to 10% of cases for the Duffy and Kidd systems by comparing phenotyping and genotyping results [6,18,20]. These findings differ from our data, which showed a much higher rate of discrepancies (32.7% and 31.7%), although the results cannot be accurately compared because other authors failed to provide information about the number of transfused RBC units or the time frame from the last transfusion.

In contrast to a number of previous studies [6,18,20,21,22], our data do not suggest the presence of additional Duffy system alleles (which are known to be associated with reduced or abolished gene expression) either in patients or in donors. This is likely due to the fact that only genotypes of the Caucasian population were tested in our study.

The phenotyping/genotyping results in a group of patients who received transfusions within a time period of 7 days before sample collection also differed from those reported earlier, particularly for the Kidd system [5,7]. In this group, a total of 24 samples in our study showed positive (2+ to 4+) results for Fya, Fyb, Jka, and Jkb, although genotypically, 14 of them were Fya- or Fyb-negative, and 14 were Jka- or Jkb-negative. Reid et al. described concordant phenotype/genotype results for 7 samples and mixed-field agglutination for 32 samples for Duffy antigens; they also described coincident results for 16 samples and mixed-field agglutination for 31 samples for the Kidd system [7].

Our data suggest the need for blood group genotyping (including the Duffy and Kidd systems), which was proposed by many authors [1,2,3,4,5,6,7,11,13,16,18,19,20,23,24,25]. Nevertheless, one should determine when genotyping should replace serology or be combined with it for patients undergoing long-lasting RBC transfusions. This is particularly important for institutions that do not perform blood group genotyping routinely. We found in our study that Duffy genotyping/phenotyping disagreements depended on the number of transfused RBC units during the last 3 months. However, it would be difficult to predict how many units would cause these discrepancies or to make specific recommendations. The limitation of our study is the small number of subjects, which complicated their grouping according to the number of transfused RBC units.

The results of our study regarding the effect of the length of time after the last transfusion on serology findings are useful in clinical practice. A significantly lower number of Duffy and Kidd phenotyping/genotyping discrepancies was found in patients who underwent their last transfusions more than 3 (p=0.01 and p=0.017, respectively) or 4 (p=0.003 and p=0.018, respectively) weeks before the sample collection. Only one disagreement for the Duffy system (compared to 3 for the Kidd system) was found when the time from the last transfusion was from 6 to 7 weeks. No discrepancies were found for either system when the last transfusion was performed more than 7 weeks before.

Our data are also in agreement with previous reports indicating that the range of agglutination in mixed-field reactions does not predict the actual antigen typing [7]. For example, 10 patients in the discrepant group with 2+ reaction for Fya and 2+ or 3+ reaction for Fyb had FY*B/FY*B genotypes. In the group with accurate results, 7 genotypically FY*A/FY*B samples also showed 2+ agglutination with anti-Fya. An exception could be made for serologic reactions with the expression of 1+. All 5 of the 1+ reactions in the Duffy system typing as well as 8 respective reactions in the Kidd typing genetically showed no allele expression. Those reactions were classified as serologically negative. More research is needed to confirm this statement.

**Conclusions**

Our study showed that Duffy or Kidd phenotyping with agglutination tests is inappropriate for patients who have recently undergone blood transfusion. Genotyping should be considered when the patient underwent transfusion less than 4 weeks before the sample collection. The group of patients who recently (<7 weeks) received RBC transfusion and require genotyping in addition to phenotyping are those who are homozygous for either Duffy or Kidd, but this cannot be ascertained using serology. Agglutination tests can be reliably used for donors and patients who have been transfused more than 7 weeks before.

Furthermore, the accurate typing of both the Kidd and Duffy systems before the first transfusion could be beneficial.

**Conflict of Interest Statement**

The authors of this paper have no conflicts of interest, including specific financial interests, relationships, and/or affiliations relevant to the subject matter or materials included.

## Figures and Tables

**Table 1:**
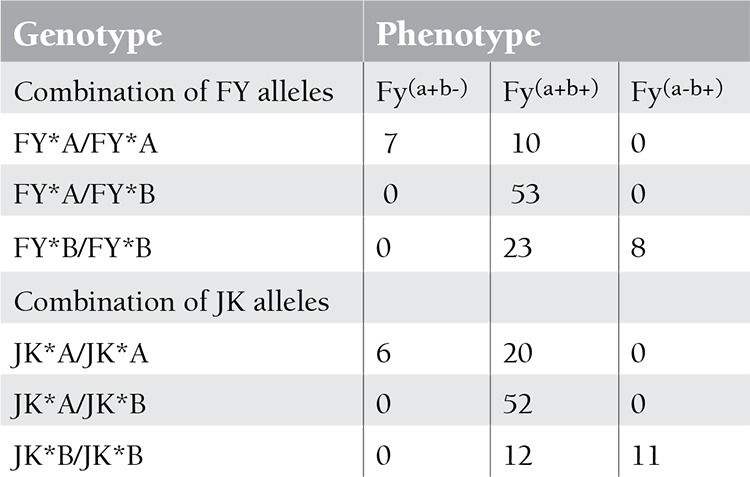
The results of Duffy and Kidd system phenotyping and genotyping in 101 multitransfused patients.

**Table 2:**
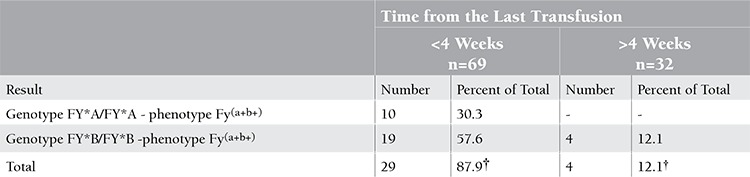
Duffy phenotyping and genotyping discrepancies and time from last red blood cells transfusion.

**Table 3:**
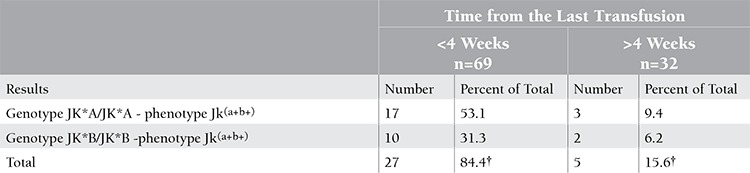
Kidd phenotyping and genotyping discrepancies and time from last red blood cells transfusion.

**Table 4:**
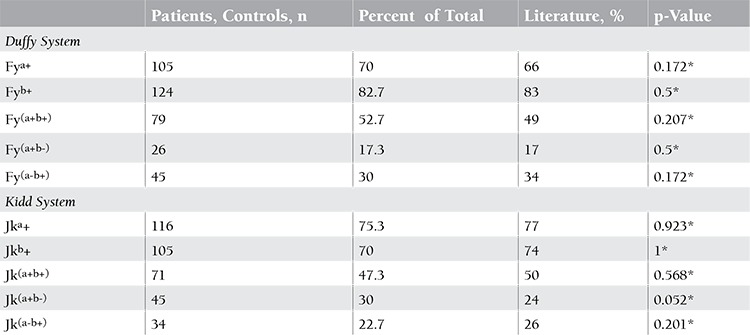
Distribution of Duffy and Kidd antigens and phenotypes.
